# FrzS Regulates Social Motility in *Myxococcus xanthus* by Controlling Exopolysaccharide Production

**DOI:** 10.1371/journal.pone.0023920

**Published:** 2011-08-19

**Authors:** James E. Berleman, Juan J. Vicente, Annie E. Davis, Sharon Y. Jiang, Young-Eun Seo, David R. Zusman

**Affiliations:** Department of Molecular and Cell Biology, University of California, Berkeley, California, United States of America; Loyola University Medical Center, United States of America

## Abstract

*Myxococcus xanthus* Social (S) motility occurs at high cell densities and is powered by the extension and retraction of Type IV pili which bind ligands normally found in matrix exopolysaccharides (EPS). Previous studies showed that FrzS, a protein required for S-motility, is organized in polar clusters that show pole-to-pole translocation as cells reverse their direction of movement. Since the leading cell pole is the site of both the major FrzS cluster and type IV pilus extension/retraction, it was suggested that FrzS might regulate S-motility by activating pili at the leading cell pole. Here, we show that FrzS regulates EPS production, rather than type IV pilus function. We found that the *frzS* phenotype is distinct from that of Type IV pilus mutants such as *pilA* and *pilT*, but indistinguishable from EPS mutants, such as *epsZ*. Indeed, *frzS* mutants can be rescued by the addition of purified EPS, 1% methylcellulose, or co-culturing with wildtype cells. Our data also indicate that the cell density requirement in S-motility is likely a function of the ability of cells to construct functional multicellular clusters surrounding an EPS core.

## Introduction

Bacteria modulate activity in response to cell density and surface cues, allowing groups of cells to accomplish more than individuals [1], [2]. *Myxococcus xanthus* is a Gram-negative soil bacterium that utilizes multicellularity during vegetative swarming, predation of prey microorganisms and, when nutrients are reduced, aggregation into fruiting bodies that contain spores [3], [4]. Motility is required for all of these functions. Unlike other surface motile bacteria such as *Bacillus subtilis* and *Proteus mirabilis*, *M. xanthus* does not produce flagella but glides on surfaces through a combination of Social (S) and Adventurous (A) motility [5], [6], [7]. The two systems can be synergistic, but confer distinct advantages depending on the culture conditions: S-motility promotes group movement on soft, nutrient rich surfaces like 0.3–0.5% agar, while A-motility functions best on firmer surfaces, like 1.5–2.0% agar [8], [9]. Conditions that promote S-motility result in smooth colony edges that lack isolated cells. In contrast, conditions that promote A-motility result in colony edges containing many individual gliding cells as well as groups.

S-motility occurs by extension and retraction of Type IV pili, but also requires extracellular polysaccharide matrix (EPS) [10], [11], [12]. EPS is rich in glucosamine and N-acetylglucosamine and has been proposed to serve as an anchor for pilus binding and retraction [13]. Isolated *M. xanthus* cells do not show S-motility on an agar surface; however, cells regain S-motility at high cell density [14]. This observation is the basis for the hypothesis that pili from one cell bind to EPS on the surface of a close neighboring cell, propelling cell movement [7], [13], [14]. However, distance may not be the only factor inhibiting S-motility at low cell densities, as the cell density requirement can be completely abolished by the addition of 1% methylcellulose [15]. These results indicate that the Type IV pili are synthesized and functional even at low cell densities.

A number of genetic loci have been identified that are required for S-motility, including a 37 gene cluster homologous to known *eps* biosynthesis genes [12], [16], [17]. Polysaccharide slime has also been implicated in A-motility, but a causal connection between polysaccharide production and A-motility has not been established. Based on recent data, A-motility has been proposed to be powered by proton motive force (PMF) and driven by distributed motor proteins that move along a helical track, creating differential drag forces that distort the cell surface and generate surface waves that push cells forward [18].

To achieve directed motility, *M. xanthus* cells need to periodically reverse. Cell reversals in *M. xanthus* involve the inversion of cell polarity so that the lagging cell pole becomes the new leading cell pole and the old leading cell pole becomes the new lagging cell pole [19], [20]. Since the pili required for S-motility are found only at the leading cell pole, precursor and regulatory proteins required for S-motility must either be transported from pole-to-pole when cells reverse or be present at both cell poles, but subject to periodic activation/inactivation by regulators [21]. For example, monomers of the major pilin subunit, PilA are localized throughout the membrane but are assembled only at the leading cell pole [22]. By contrast, FrzS, an S-motility protein controlled by the *frizzy* (Frz) chemosensory system, periodically translocates from pole to pole during cellular reversals [23], [24]. It is therefore of interest to determine the role of FrzS in S-motility and the reason for its periodic pole-to-pole translocation.

FrzS contains two principal domains: an N-terminal pseudo-receiver domain that lacks the critical aspartyl residue that is typically phosphorylated in two component signaling systems, and a C-terminal coiled-coil domain, predicted to be involved in the dynamic assembly or disassembly of protein complexes [25]. The coiled-coil domain of FrzS promotes oligomerization when overexpressed in *E. coli*. [24]. Although FrzS lacks the canonical sites for activation of the receiver domain by a cognate kinase, two receiver domain point mutations result in mislocalization (Y102A, H92F) of the protein and loss of function [26]. These domain structures suggested that FrzS might function as an intermediary, transducing signals from the Frz chemosensory system to activate pilus assembly at the leading cell pole [23], [24]. Unexpectedly, the results presented in this study show that FrzS, rather than being a regulator of pilus production, is actually a regulator of EPS production.

## Results

### *frzS* mutant cells show restored S-motility when mixed with wildtype cells

**Figure 1:**
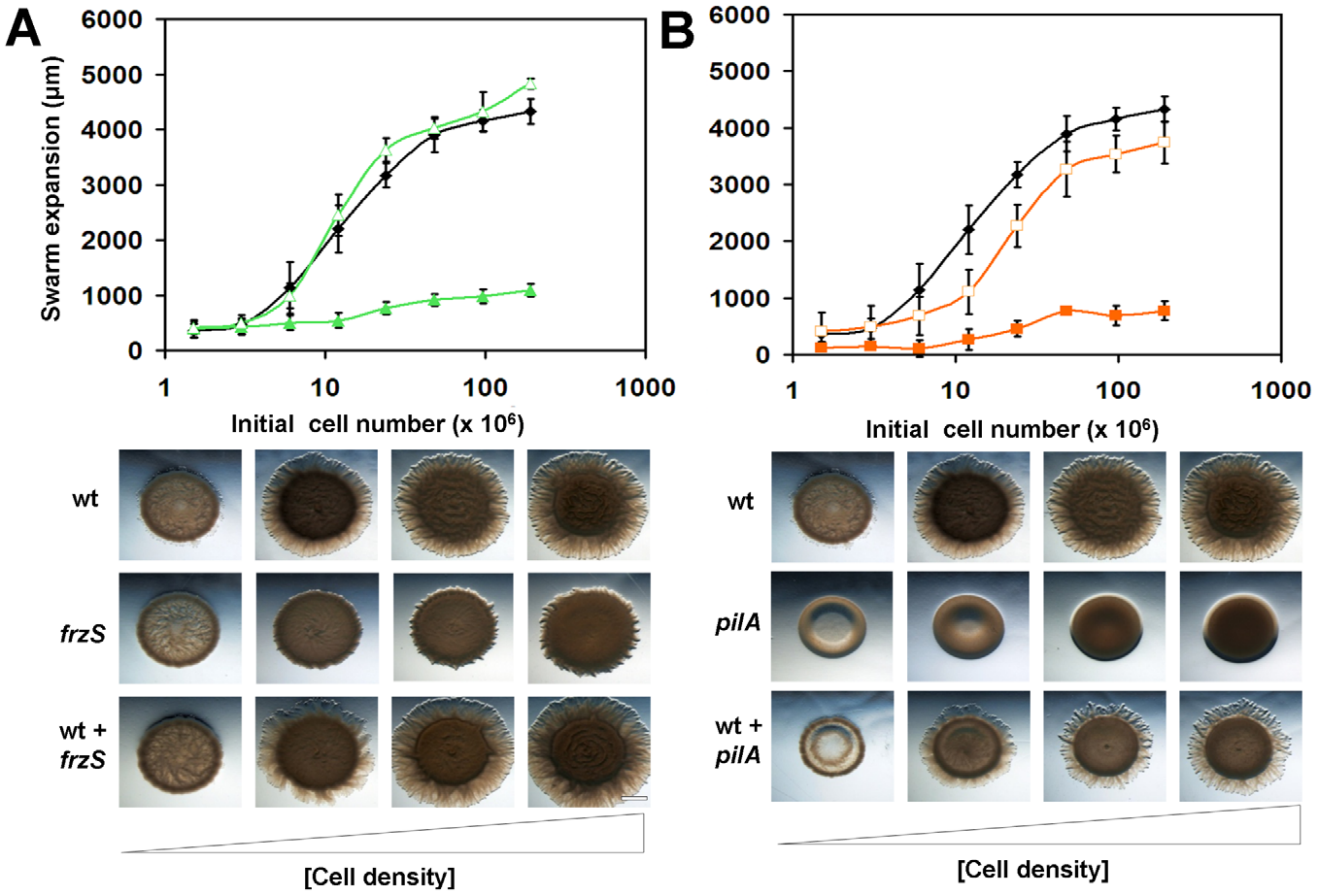
Quantitative social motility assay. Serial dilutions of mid exponential-phase cultures ranging from 2×10^6^ to 2×10^8^ cells/assay were prepared either in mono-cultures or co-cultures, then aliquoted onto 0.5% agar CYE plates. Swarm expansion was imaged and measured after 24 h incubation at 32°C. (A) Cell density-dependent social motility was assayed in wildtype (black ♦), *frzS* (green ▴) and a wildtype+*frzS* co-culture (green ▵), with the error bars representing one standard deviation. Corresponding images below show wildtype swarms, defective *frzS* swarms with small abortive flares, and full recovery of motility in the co-culture. (B) Analysis of wildtype (black ♦), S-motility defective *pilA* (orange ▪) and partial recovery of a wildtype+*pilA* co-culture (orange □) with corresponding images from the even number data points shown below. Scale bar is 1 mm.

S-motility in wildtype *M. xanthus* strain DZ2 is best observed when cells are incubated at high cell density on nutrient rich, 0.5% agar CYE plates, as A-motility does not function under these conditions. On this medium, *M. xanthus* cells glide away from the initial inoculum in a cell density dependent manner, producing thin spreading swarms. In most cases, capturing an image by 24 h is sufficient to demonstrate normal S-motility or S-motility defects. To quantify S-motile swarming, we prepared two-fold serial dilutions of exponential phase cultures, and then spotted 3 µl aliquots of cells onto 0.5% agar CYE plates at cell densities ranging from 2×10^6^ to 2×10^8^ cells. We measured the size of the initial spots and the swarm diameters after 24 h at 32°C to determine swarm expansion as a function of initial cell density. The results are shown in Fig. 1. In this assay, wildtype cells showed cooperative swarming as cell densities were increased: (i) poor swarming was observed at low cell densities; (ii) as cell densities were increased, there was a sharp increase in the rate of swarm expansion; (iii) at the highest cell densities, a maximum swarm expansion rate was achieved (see Fig. 1). The maximum swarm expansion rate for wildtype cells was 4.3 mm/day, and the cell number at which half the maximum swarm expansion occurs was 2×10^7^ cells. We consider this number to be important for quantifying cooperative social swarming as any decrease in cooperation should shift the curve to the right increasing the number of cells required for rapid swarm expansion.

In contrast, S-motility mutants like *pilA* (DZ4469) showed little swarm expansion, even at high initial cell densities, and exhibited smooth colony edges (see Fig. 1B). Interestingly, co-culturing wildtype with *pilA* (1∶1) showed an intermediate swarm expansion rate of 3.0 mm/day, indicating that wildtype cells are not inhibited by the presence of non-motile *pilA* cells in the swarm (see Fig. 1B). The behavior of the wildtype-*pilA* co-culture indicates that *pilA* cells are not counted as part of the functional swarming group, i.e., an initial cell population of 2×10^7^ cells in which 50% are wildtype behaves the same as an initial cell population of 1×10^7^ cells that are 100% wildtype. In contrast, *frzS* swarms (DZ4335) differed from both wildtype cells and *pilA* cells. Instead of smooth colony edges like the *pilA* mutants, *frzS* swarms showed wrinkled colony edges, consisting of tiny flares emerging from the swarms (see Fig. 1A, lower panel). After 24 h, the net swarm expansion was extremely small, only about 1 mm. However, co-culturing of wildtype and *frzS* cells (1∶1) resulted in a swarm expansion rate similar to wildtype levels, 4.3 mm/day. The result that wildtype-*frzS* mixed swarms expand at the same rate as wildtype alone is consistent with and suggestive of the possibility that wildtype fully rescues the swarming defect of the *frzS* mutant. This complementation was not observed in other motility mutants, including *pilT* (DK10409), *tgl* (DZ4191), *cglB* (DZ4477), *mglA* (TM12), *frzF* (DZ4483), and *difA* (YZ601) strains (data not shown). All of these mixtures showed a phenotype similar to wildtype cells mixed 1∶1 with buffer, indicating that non-motile cells in this assay do not inhibit wildtype swarming, and that the phenotype of wildtype-*frzS* co-cultures can only be due to increased activity of one or both strains.

**Figure 2:**
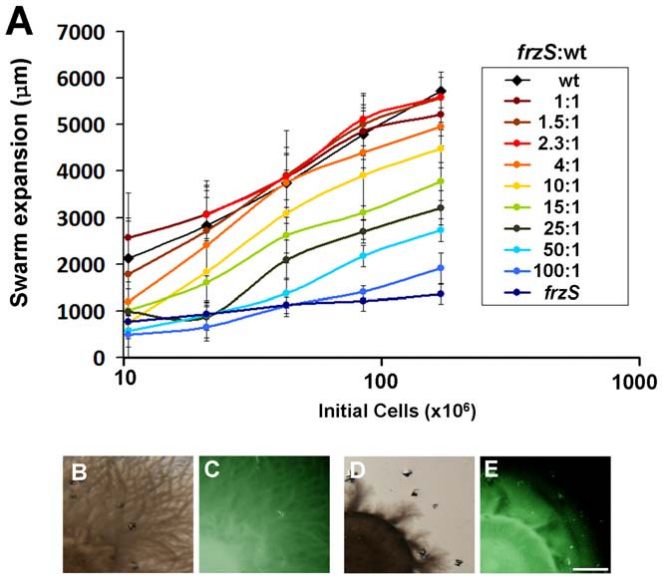
Complementation of the *frzS* Social motility defect. (A) Swarm expansion data for *frzS*-wildtype co-cultures at the ratios listed. (B,C) Transmission and fluorescence stereomicroscopy of 1∶1 co-cultures of wildtype with *frzS^Y102A^::gfp* and (D,E) *ΔfrzS* with *frzS^Y102A^::gfp* showing migration of the GFP-labeled cells corresponding to the colony edge. Scale bar is 1 mm.

The extracellular complementation by wildtype is robust as up to a 4∶1 *frzS* to wildtype cell ratio resulted in maximal swarm expansion (see Fig. 2). Higher ratios of *frzS* to wildtype cells reduced swarm expansion incrementally, with a 9∶1 ratio yielding a phenotype similar to a 1∶1 *pilA*-wildtype mixture, and a 100∶1 ratio required to yield a phenotype similar to *frzS* alone. To further determine if *frzS* cells migrate with wildtype cells from the initial inoculum through to the colony edge in the 0.5% agar S-motility assay, we analyzed co-cultures of wildtype and *frzS* with DZ4538, a *frzS^Y102A^*::*gfp* mutant that expresses a C-terminal Green Fluorescent Protein (GFP) tag [26]. We examined these co-cultures with fluorescence stereo microscopy and imaged the colonies after 24 h incubation (see Fig. 2B–E). The images show that indeed the *frzS^Y102A^*::GFP strain was motile in the co-culture since fluorescence was visible from the colony center out to the periphery in both mixtures.

### Isolated *frzS* cells are motile in 1% methylcellulose

**Figure 3:**
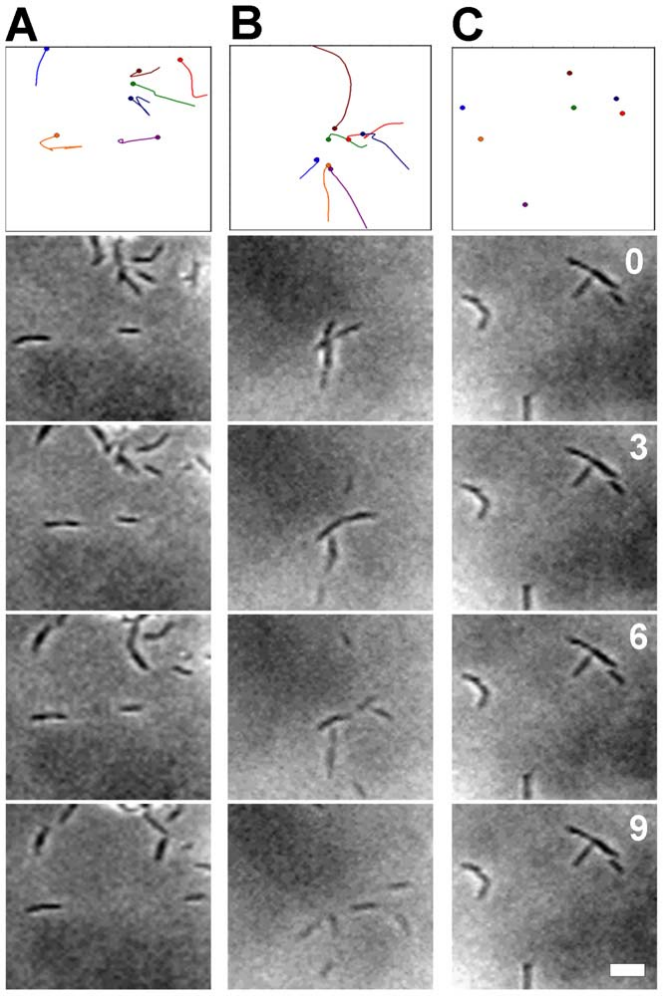
Motility assay in methylcellulose. Mid-exponential phase cells were resuspended in 1% methylcellulose on glass slides and S-motility observed by time-lapse microscopy. Images show 7 cell tracks for (A) *frzS* mutant; (B) wildtype strain DZ2: and (C) *pilT* mutant over 9 min (Top). Images at 0, 3, 6, and 9 min are shown below. Cells were tracked in three independent experiments each with *frzS*, wildtype and *pilT*, but movement was only observed with *frzS* and wildtype DZ2. Scale bar is 5 µm.

Previous studies of FrzS led to the hypothesis that FrzS regulates S-motility by activating PilT ATPase retraction at the leading cell pole [20], [24]. However, the ability of *frzS* cells to show S-motility swarming when co-cultured with wildtype cells indicates that they may harbor a defect other than pili function. We used the 1% methylcellulose assay developed by Sun *et al.* to analyze the behavior of isolated *frzS* cells [15], [27]. Cells were harvested from exponential phase cultures, mixed with 1% methylcellulose and observed immediately with phase contrast microscopy. By tracking cells at 1 min intervals for 20 min, isolated *frzS* cells were observed to show motility behavior similar to wildtype (see Fig. 3). Thus, *frzS* cells produce functional Type IV pili that can propel cells forward. In contrast, isolated *pilT* cells were completely non-motile in 1% methylcellulose.

### *frzS* cells are defective in EPS production and cell-cell agglutination

**Figure 4:**
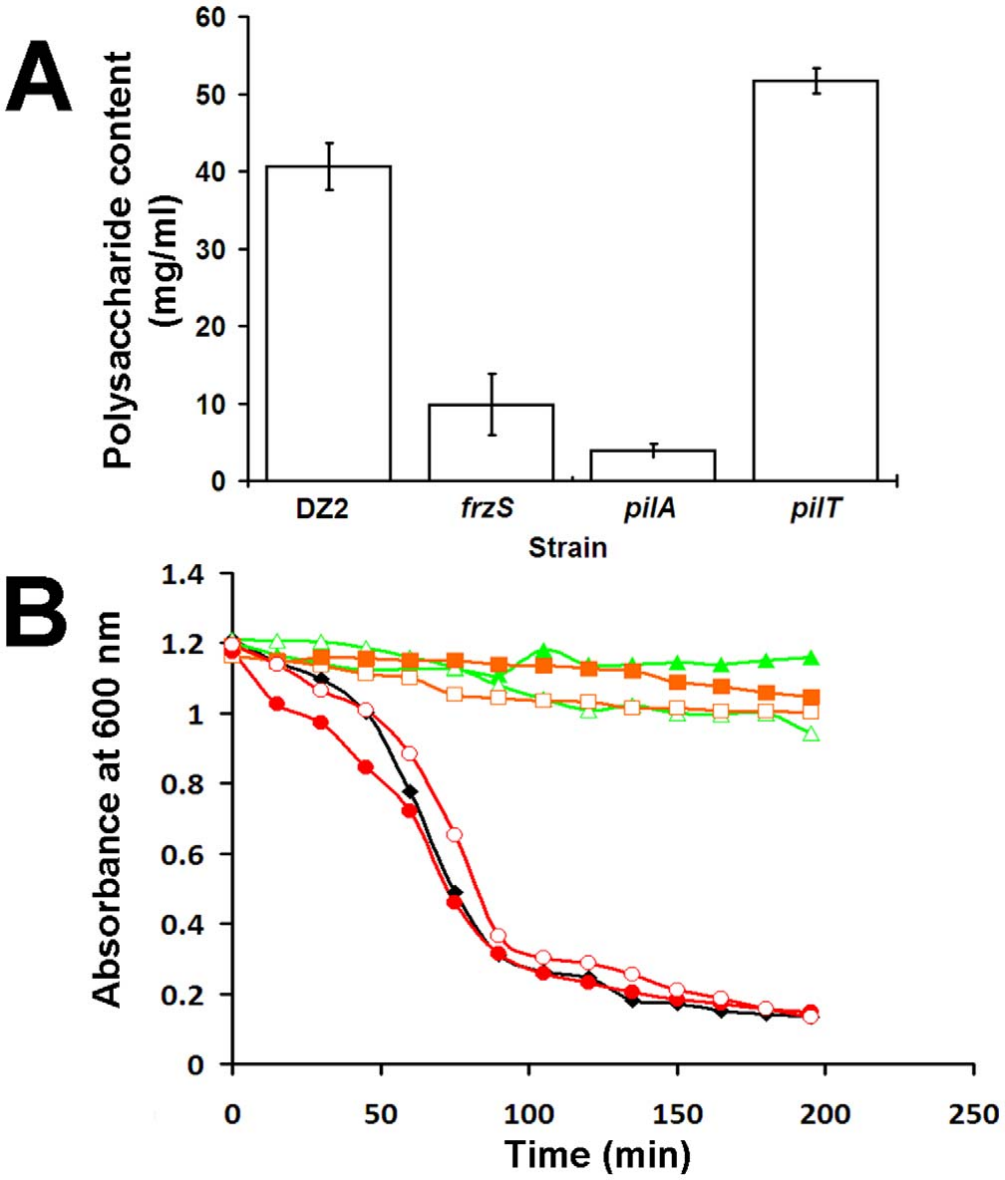
EPS analysis and agglutination assay. (A) Congo red dye was added to mid-exponential phase cultures of the given strains, cells were centrifuged and the resulting supernatants analyzed in a spectrophotometer at 490 nm. Values were normalized to wildtype binding. An increase is indicative of more Congo red bound by the cells and increased EPS production, such as observed in *pilT*. *pilA* and *frzS* cells show reduced EPS production. Data is from three independent trials. (B) Cells were also analyzed with agglutination assays performed on wildtype (black ♦), *frzS* (green ▴), *pilA* (orange ▪), and *pilT* (red •), and a 1∶1 mixture of wildtype with the mutant (open symbols). In wildtype, absorbance at OD_600_ at the top of a cuvette decreases over time as cells agglutinate and settle to the bottom of the cuvette. *pilA* and *frzS* inhibit agglutination by wildtype, whereas *pilT* was observed to behave similar to wildtype. Data shown is representative of three trials.

Since examination of *frzS* cells showed evidence of functional type IV pili, we hypothesized that *frzS* might be defective in EPS production or secretion. We therefore examined the *frzS* mutant for EPS production using Congo red dye, previously shown to bind to *M. xanthus* EPS [28]. Cells were harvested from exponentially growing cultures, mixed with Congo red dye and centrifuged. Unbound dye was measured at 490 nm, and polysaccharide content was determined relative to a cellulose standard curve. Figure 4A shows that *frzS* cells bind Congo red dye but only at 25% of wildtype levels. *pilA* mutants, which have a more severe defect in EPS production, bound EPS at 10% of wildtype levels. In contrast, a *pilT* mutant, defective at pili retraction, overproduces EPS at a level 127% of wildtype. These data indicate that *frzS* is defective in EPS production, but do not indicate what distinguishes *frzS* from the other S-motility mutants.

EPS is also required for cell-cell agglutination, which is observed when *M. xanthus* cells in suspension bind to each other and settle to the bottom of a cuvette [29], [30]. In wildtype cells, this is observed as an exponential drop in absorbance at 600 nm (see Fig. 4B). However, *frzS* cells were defective in agglutination (see Fig. 4B) and remained suspended in liquid similar to EPS deficient mutants like *pilA*. These results differ from previously published agglutination data for a *frzS* insertion mutant (DZ4219) [8]. We therefore retested both mutant strains for agglutination, EPS production and social motility (see Fig. S1). In our hands, both *frzS* mutant strains were found to be defective in these assays. Interestingly, when either *frzS* or *pilA* were mixed in a 1∶1 ratio with wildtype cells, the co-culture showed significantly reduced agglutination relative to wildtype (see Fig. 4B). This surprising result indicates that EPS defective cells interfere with the ability of wildtype cells to form aggregates necessary for agglutination. This was not a cell density effect since wildtype cells by themselves did not show reduced agglutination when cell densities were reduced by half (data not shown). The *pilT* mutant, which overproduces EPS, agglutinates more rapidly than wildtype and stimulates wildtype agglutination (see Fig. 4B). These data indicate that *frzS* cells are not only defective in EPS-dependent agglutination, but they are also not much better than *pilA* cells at agglutination, even when mixed with wildtype.

### Purified EPS can restore S-motility to *frzS* mutant cells

**Figure 5:**
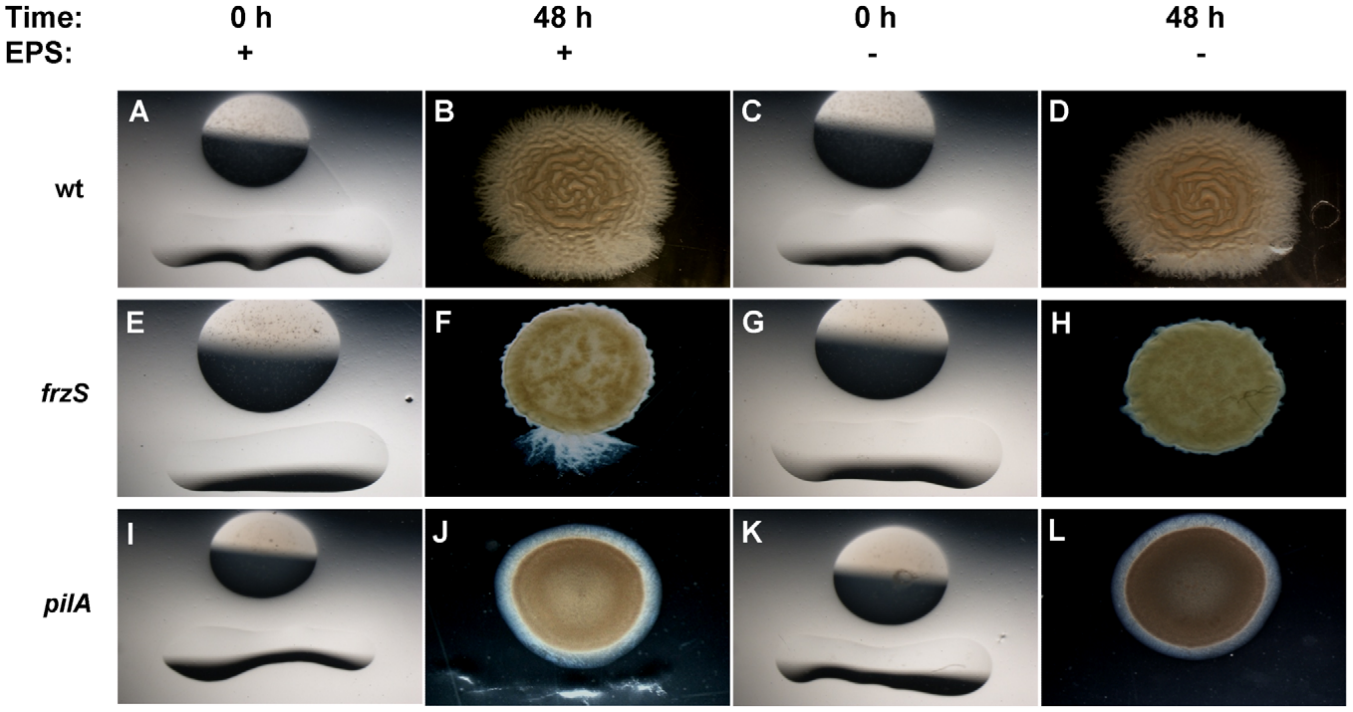
Complementation of S-motility with purified EPS. Washed cells of wildtype (A–D), *frzS* (E–H), and *pilA* (I–L) were spotted onto 0.5% agar CYE plates adjacent to a thin line of either an aliquot of EPS purified from wildtype (left two columns) or a buffer control (right two columns). Images were captured at 0 and 48 h, showing stimulation of motility in wildtype and *frzS* cells, but not *pilA* cells. Stimulation of motility can be observed as downward outgrowth from the colony. Images show representative images from multiple trials.

To determine whether exposure to EPS is sufficient for the restoration of S-motility to *frzS* swarms, we purified EPS from wildtype cells and pipetted this preparation as a linear streak adjacent to spots of wildtype, *frzS*, or *pilA* cells on 0.5% agar plates (Fig. 5). In this assay, wildtype cells increased their swarm expansion as they contacted the zone containing EPS. Significantly, the *frzS* cells, which were otherwise non-motile, also showed a burst of swarming but only in the EPS zone. The *pilA* mutant did not respond to the EPS nor did *frzS* cells exposed to a control buffer (Fig. 5). This indicates that purified EPS is sufficient to restore S-motility to *frzS* swarms, and that *frzS* is not required for pili retraction during soft agar swarming.

Additionally, we purified EPS from wildtype, *pilT*, *pilA* and *frzS* cells and tested these EPS preparations for their ability to stimulate *frzS*, *pilA*, and *difA* mutant swarming (see Table 1). All of these strains produced some EPS and they all stimulated S-motility in the *frzS* mutant. However, none of the EPS preparations were able to stimulate motility in the *pilA* or *difA* mutants. This is consistent with previous observations that showed *pilA* and *difA* mutants have S-motility defects in both pili function and EPS production [31], [32].

**Table 1:** EPS complementation.

Test strain	EPS Source				
	DZ2	*pilT*	*frzS*	*pilA*	Buffer
*frzS*	++	++	++	+	−
*pilA*	−	−	−	−	−
*difA*	−	−	−	−	−

EPS was purified from several strains and stimulation of motility assayed in *frzS*, *pilA* and *difA*.

### Multicellular localization of EPS

**Figure 6:**
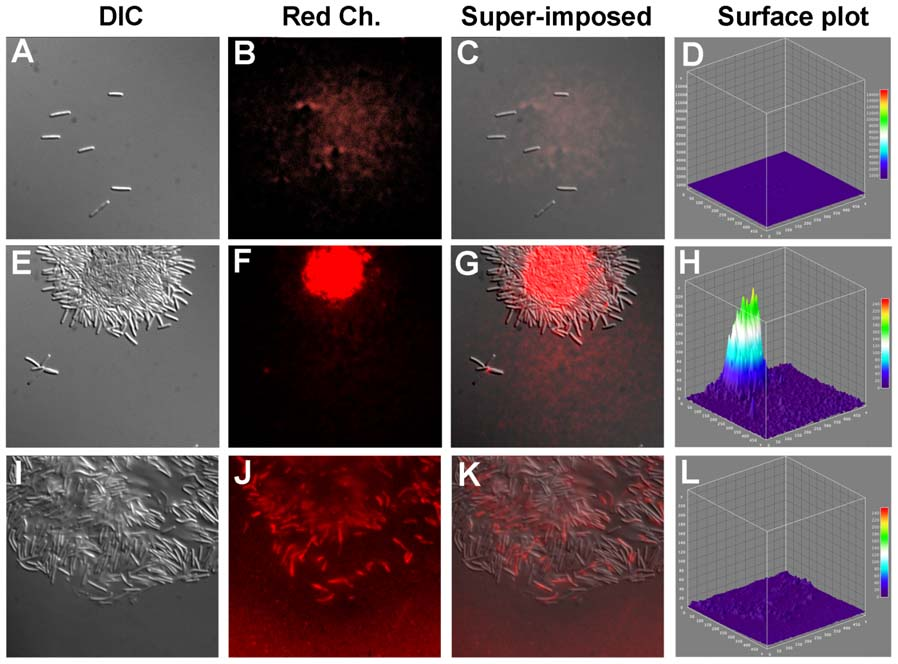
Fluorescence microscopy analysis of EPS production. DIC and fluorescence analysis of (A–D) wildtype *M. xanthus* cells from a liquid culture. (E–H) Wildtype *M. xanthus* cells after 4 h incubation with a solid surface in submerged culture, showing the formation of small and large rosette structures with centralized WGA-TR staining. (I–L) *frzS* cells after 4 h incubation with a solid surface, showing disorganized clustering and dispersed low levels of WGA-TR staining. Each data set depicts from left to right: DIC, red channel fluorescence, superimposed overlay, and surface plot of signal intensity.

To determine whether EPS production has an impact on cellular organization, we examined wildtype *M. xanthus* cells harvested from exponential phase cultures and cells 4 h after incubation on a plastic surface in submerged culture with Differential Interference Contrast (DIC) and fluorescence microscopy (see Fig. 6A–L). Liquid grown cells were dispersed and stained poorly with wheat germ agglutinin conjugated to a Texas red fluorophore (WGA-TR). However, after surface incubation, wildtype cells were observed as both isolated cells and tightly packed cell clusters organized around a central bundle that stains brightly with WGA-TR (see Fig. 6E–H). Most cells were observed in large clusters of up to 10^3^ cells with a high intensity feature that localized to the center of the cell clusters rather than to individual cells. Interestingly, very small clusters of cells (for example, see panel 5E) showed 4–5 cells radially organized around a small WGA-TR binding feature (see panel 5F), reminiscent of rosette structures observed in *Caulobacter crescentus* [33]. In contrast, the *frzS* cells showed fewer clusters after surface incubation, and these clusters were more disorganized than those of wildtype (see Fig. 6I–L). WGA-TR staining of *frzS* clusters showed reduced and decentralized staining, with the stain more likely to co-localize with single cells, rather than at distinct multicellular foci.

### Construction and analysis of EPS biosynthetic mutants

**Figure 7:**
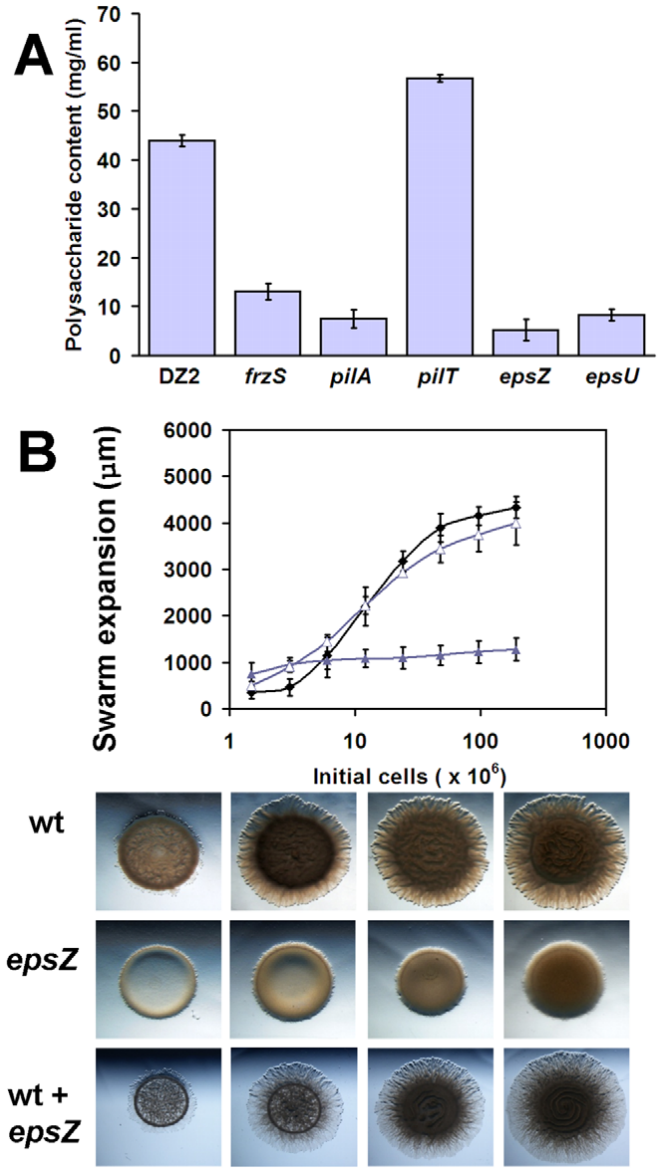
EPS mutant analysis. (A) Congo red analysis of EPS extracts from the given strains. (B) Quantitative social motility with wildtype (black ♦), S-motility defective *epsZ* (purple ▴), and a 1∶1 co-culture of the two strains (purple ▵) showing increased swarming relative to *epsZ* alone, with corresponding images shown below.

To compare the observed *frzS* phenotype to that of mutants in EPS production, we constructed mutations in several of the genes predicted to be required for EPS production. We targeted mxan_7415 (*epsZ*), and mxan_7422 (*epsU*), genes that are predicted to catalyze glycosyl transferase reactions critical for EPS biosynthesis and mxan_3227 and mxan_3229, genes predicted to provide the same function in the production of capsular polysaccharide (CPS) [12], [34]. Insertion mutants were constructed in theses strains as described previously [24]. Analysis of these strains indicates that *epsU* (DZ4830) and *epsZ* (DZ4831) are defective in EPS production (Fig. 7A), whereas no defect was detectable in the mxan_3227 and mxan_3229 loci (data not shown). Congo red dye-binding assays showed that *epsZ* and *epsU* mutants contained polysaccharide less than or equal to that found in *pilA* extracts.

**Figure 8:**
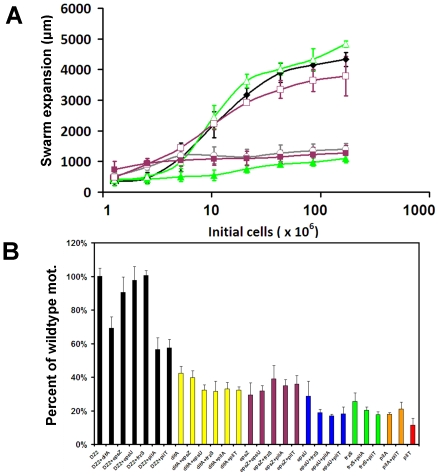
Pathway analysis. (A) Quantitative S-motility assay examining wildtype (black ♦), *frzS* (green ▴), wildtype+*frzS* (green ▵), *epsZ* (purple ▪), wildtype+*epsZ* (purple □ ) and *epsZ*+*frzS* (gray ○). (B) Combinatorial quantitative social motility analysis of DZ2, *difA*, *epsZ*, *epsU*, *frzS*, *pilA*, and *pilT* strains. Mono-culture and co-culture combinations of these strains are shown, indicating that *frzS*, *epsZ*, and *epsU* are in the same functional pathway as they are both complemented by the presence of wildtype, but are not able to complement each other.

The *epsZ* mutant was defective in S-motility, but showed no defect in swarm expansion when incubated in a 1∶1 co-culture with wildtype strain DZ2 (Fig. 7B). Since *frzS* and *epsZ* mutants could both be complemented by wildtype cells, we hypothesized that if they were functioning within the same pathway, then they should not be able to complement each other. Alternatively, if *frzS* and *epsZ* effect different aspects of S-motility, then they should be able to complement each other and restore S-motility. We therefore analyzed S-motility in different combinations of mixed cultures to determine the effect of co-culturing cells (Fig. 8). For example, a 1∶1 *frzS*-*epsZ* mixture showed a slight elevation in S-motility relative to each strain in mono-culture, but motility levels were still low, at less than 30% of wildtype levels and therefore not statistically significant (see Fig. 8A). We expanded this analysis to include mixtures with wildtype DZ2, *difA*, *epsZ*, *epsU*, *frzS*, *pilA* and *pilT* (see Fig. 8B). All strains were analyzed in mono-culture for their maximum swarm expansion, ranging from 42% (*difA*) to 12% (*pilT*) of wildtype levels. Next, we examined all 21 possible co-culture combinations, to determine if it is possible to identify two S-motility defective mutants that, when combined, show fully functional S-motility. Our results are summarized as follows: (i) Mixtures of wildtype with *frzS*, *epsZ* and *espU* showed wildtype levels of swarming. (ii) All strains mixed with *epsZ* showed motility levels equivalent to the most proficient of the two strains. (iii) Strains with the lowest swarm expansions, *pilA* and *pilT* showed a slight increase in swarm expansion when mixed. (iv) Most other combinations showed swarm expansions that were intermediate to the individual mono-culture phenotypes. This analysis indicates that there are no clear complementation groups among these S-motility defective strains, but that in certain cases the more defective S-motility mutant can be partially complemented by the more motile strain. The EPS^+^ phenotype alone is not enough for complementation in this assay (e.g. *frzS* mixed with *pilT*), and the EPS producing strain must also be capable of moving across the surface. These results indicate that EPS^−^ cells are not able to bind and glide away with EPS from donor EPS^+^ cells.

## Discussion

The data presented here suggest a new model for the role of FrzS and cell density in *M. xanthus* S-motility. We began with the development of a quantitative S-motility assay that allowed us to analyze the contribution of different cell densities and S-motility genes on multicellular swarming. These studies showed that *frzS* cells are S-motile when mixed with wildtype cells, despite the inability of *frzS* to function in mono-culture. This behavior phenocopies the EPS mutants, *epsZ* and *epsU*. Additionally, isolated *frzS* cells are S-motile in 1% methylcellulose. In contrast, mutants defective in pili function do not show restored motility in co-cultures with wildtype cells. These results show that, rather than regulating extention/retraction of the Type IV pili, FrzS functions in the regulation of EPS biosynthesis and/or secretion. *frzS* cells are able to produce low levels of EPS, since we could purify EPS from *frzS* cultures and, when concentrated, use this EPS to rescue S-motility swarming in the same *frzS* mutant (Table 1). It is possible that FrzS regulates secretion of EPS, but since EPS secretion, polymerization, and translocation may be carried out by the same enzyme, all of these functions may be affected [35]. Since FrzS was previously shown to form polar clusters that move from pole-to pole when cells reverse [20], it is possible that FrzS directly interacts with EPS secretion channels along the cell membrane or at the cell poles, activating them in response to a signal received, presumably through the Frz pathway. Another possibility is that FrzS is involved in sensing cell clusters. Cell clustering is weak and disorganized in a *frzS* mutant, and it is not yet clear if this disorganized state causes the defect in EPS production, or is the result of it.

The data presented here also suggest major revisions in our understanding of S-motility in *M. xanthus*. Previously, it was shown that S-motility has a cell density requirement, which was interpreted to be a function of cell-cell distance [14]. Thus, isolated cells showed no S-motility but cells that were making cell-cell contact or in the immediate vicinity of another cell became S-motile. It was suggested that the presence of neighboring cells coated with EPS (within a 5 µm radius) provided an anchor for pilus binding and retraction and therefore a basis for cell movement. Our data support a different hypothesis. We suggest that secreted EPS may be the usual anchor for the Type IV pili, rather than the presence of neighboring cells *per se*. Our conclusions are based on the following: (i) Cells showed full S-motility in the absence of neighboring cells when immersed in 1% methylcellulose; and (ii) Cells at high cell density showed maximal S-motility swarming, while cells at a lower cell density, but still in intimate contact, showed almost no S-motility. For example, we examined S-motility at cell densities ranging from 0.25 to 25 cells/µm^2^. Confluent cell coverage of the area was observed at all of these cell densities; indeed the average *M. xanthus* cell size is 5 µm^2^, indicating that cells were tightly packed. At 0.25 cells/µm^2^, swarm expansion was extremely low, even though cells were in contact; if cell distance were the critical factor, then the minimum cell density necessary for S-motility should have been much lower. The observation of organized multicellular rosettes ranging from 10^1^–10^3^ cells, containing a core of intense WGA-TR signal indicative of EPS, suggests that the cell density requirement in S-motility may reflect the point at which cells are able to successfully form these higher order structures. The formation of higher order structures in *M. xanthus* is not surprising, given that this organism is distinctive for its ability to form fruiting bodies containing 10^6^–10^7^ cells [36], [37].

The observed extracellular rescue of *frzS* Social swarming by either S-motility^+^ cells or purified EPS, is reminiscent of previous work demonstrating conditional, or stimulatable motility in *M. xanthus*. Previously, it has been shown that some motility mutants of *M. xanthus* can be rescued by the presence of motility proficient cells [38], [39]. In the case of stimulatable defects in A-motility, five mutants *cglA-E*, form a single gene cluster on the *M. xanthus* chromosome. One locus has been shown to have stimulatable S-motility, the *tgl* locus. We examined several *cgl* and *tgl* strains during the course of this study and were unable to detect any evidence of stimulation of motility using the soft agar quantitative S-motility assay (data not shown). These strains, both *cgl* and *tgl*, were successfully stimulated in a cross streak assay on hard agar with either wildtype DZ2 or a non-motile *mglA* mutant as the donor strain in the cross-streak. In the case of *cgl*, this is not surprising, as A-motility does not function efficiently on soft agar. However, in the case of *tgl* it is surprising that motility rescue occurs only under hard agar conditions. Tgl stimulation is thought to occur through direct cell-cell transfer of protein, it is possible then that increased production of EPS under Social swarming conditions inhibits transfer between cells. Direct cell-cell transfer of lipid and protein is still a new field of study; it will be important for future studies to account for how regulation of other extracellular structures (such as EPS or S layers) impact the ability of cells to communicate with direct transfer mechanisms [40], [41].

The exact molecular function of the FrzS protein remains unresolved, specifically in terms of upstream and downstream partners. These data also bring up the intriguing possibility that EPS biosynthesis and secretion may be more complex than previously thought, and one future question is to determine how FrzS interacts with either the EPS biosynthesis proteins or other regulatory pathways in *M. xanthus*. It is also unclear what the signal input is for FrzS. Its dynamic change in localization during cellular reversals implies some connection, either direct or indirect, to the Frz pathway that regulates cell reversals. The output of the Frz system likely involves FrzZ, but it is unclear if there is any FrzZ-FrzS interaction. In addition, the impact of FrzS on the EPS production of cells indicates that there may be an association between the FrzS protein and the Dif signaling pathway [32], [42]. However, the pleiotropic nature of *dif* mutants makes it difficult to say for certain if these pathways are connected. Further experiments are required to resolve these possibilities.

As a social organism, *M. xanthus* is susceptible to dynamic population shifts, such as mutations that give rise to new cooperative or cheater phenotypes [43], [44]. One example of this occurs in fruiting body formation, where certain sporulation defective mutants reap the benefits of fruiting body formation when co-cultured with sporulation proficient strains. When subjected to cycles of nutrients and starvation, this can lead to a population crash when the percentage of cheater cells rises above a critical threshold that prevents community wide sporulation and both cell lines lose viability [45]. In the case of social swarming, future experiments will be required to determine if the *frzS* phenotype is indicative of “cheating” and if this would lead to dramatic changes in swarming capacity over time through differential growth of *frzS* and the parent strain.

## Methods

### Strains and growth conditions

The strains we worked with are listed in Table 2. Each strain was inoculated in a glass side-arm flask with 25 ml liquid CYE with a loose cotton cap for oxygen exposure. Cultures were grown with shaking at 32°C for 24–48 hours. When the cell densities reached approximately 100–150 Klett units (KU) cultures were harvested, and precise measurements of cell densities (via a Klett-Summerson photoelectric colorimeter) recorded.

**Table 2:** Strains used in this study.

Strain	Genotype or relevant properties	Source/Reference
DK10409	DK1622 Δ*pilT*	Wu *et al* (1997) [46]
DZ2	Wild type	Campos and Zusman (1975) [47]
DZ4191	DK1253 *tgl-1*	Hodgkin and Kaiser (1979) [48]
DZ4219	DZ2 *frzS::pZOrf6*	Ward *et al* (2000) [24]
DZ4335	DZ2 Δ*frzS*	Ward *et al* (2000) [24]
DZ4469	DZ2 Δ*pilA::tet*	Vlamakis *et al* (2004) [49]
DZ4477	DZ1622 *cglB::mariner*	Youderian *et al* (2003) [50]
DZ4483	DZ2 Δ*frzF*	Bustamante *et al* (2004) [51]
DZ4538	DZ2 *frzS*(Y102A)-*gfp*	Mignot *et al* (2005) [20]
DZ4830	DZ2 *epsU::pGEM*	this study
DZ4831	DZ2 *epsZ::pGEM*	this study
TM12	DZ2 Δ*mglA*	Mauriello *et al* (2010) [7]
YZ601	DK1622 Δ*difA*	Xu *et al* (2005) [52]

### Methylcellulose assay

Methylcellulose-based motility assays were performed as described previously [27]. 1 µl of cells was added to 6 µl of 1% methylcellulose and cell movement monitored through time-lapse microscopy, and images were captured every 60 sec for 20 min. Cell tracking was performed with NIH ImageJ and graphed with Microsoft Excel.

### Quantitative social motility assay

After inoculation and growth to 100–150 KU, cultures were centrifuged in a Sorvall® RC-5B at 8000 rpm for 10 minutes. Cell pellets were resuspended in 10 mM MOPS buffer to a final density of 8000 KU. Eight serial dilutions were prepared in 1∶1 steps with buffer. For each strain, 3 µl of cells at each cell density was aliquoted onto 0.5% CYE agar plates sequentially. To measure social motility in co-cultures, resuspensions were mixed 1∶1 to a total cell density of 8000 KU, followed by serial dilution. For both the mono-culture and co-culture assays, the swarming phenotypes were examined under a Nikon SMZ1500 stereo microscope at 10× magnification. The initial diameter of each aliquot was measured using imaging software NIS-Elements BR Version 3.1 and recorded onto a Microsoft Excel Spreadsheet. The plates were incubated for 24 h at 32°C and the swarm diameter measured again as an average of vertical, horizontal and diagonal transects. The total cell movement was given as the change from the final diameter minus the initial diameter. This assay was repeated a minimum of three times for each mono- or co-culture.

### EPS purification

*M. xanthus* cultures were inoculated in 25 ml CYE broth and incubated until they reached cell densities of 150–200 KU. The cultures were then harvested and the cell pellet resuspended in 25 ml of TNE buffer (100 mM Tris pH 7.5, 100 mM NaCl, 5 mM EDTA). The cell suspensions were sonicated in four 15 s pulses. Sodium Dodecyl Sulphate (SDS) was added to a final concentration of 0.1% and the cell extracts agitated for 15 min. The extracts were centrifuged for 10 min at 8,000 rpm and the pellets washed twice with 25 ml TNE+SDS. The pellets were then washed twice with 10 ml of TNE to remove any remaining SDS. The pellets were resuspended in 1 ml of 10 mM MOPS buffer and stored at −80°C until used.

### EPS complementation assay

Cultures were prepared as before to a final cell density of 500 KU. 0.5% agar CYE plates were prepared and 6 µl aliquots of the 500 KU cell suspension were spotted onto the agar using a micropipette., A 6 mm line of EPS extract or buffer control was added 4 mm below this spot using 4 µl of extract. Plates were incubated at 32°C for 48 h. They were examined under a Nikon SMZ1500 stereo microscope at 100× magnification. Images were captured at 0, 24 and 48 h and analyzed as before.

### Mutant construction

Insertion mutants in *epsZ* and *epsU* were made, as described previously, using primers to amplify a 600 bp internal region of the gene of interest, followed by cloning into pGEM vector, and selecting for integration into the *M. xanthus* chromosome using kanamycin resistance on appropriate selective media [24].

### Fluorescence microscopy

A Wheat Germ Agglutinin-Texas Red (WGA-TR) conjugate was used to detect the presence of *M. xanthus* polysaccharides. Exponential phase cultures were harvested and washed in fresh CYE. 100 µl aliquots were added to plastic 96 well plates and incubated at 32°C for 4 h. Aliquots of liquid phase cells and surface-exposed cells were mixed with WGA-TR and incubated with cells for 30 min prior to imaging. Colonies were imaged with a Nikon SMZ1500 for stereomicroscopy and cells were imaged with a Nikon Eclipse 80i using differential interference contrast (DIC) or fluorescence microscopy. Image analysis was performed with NIS Elements and NIH ImageJ.

## Supporting Information

Figure S1 **Comparison of** ***frzS*** **mutants.** (A) From left to right, images show swarm expansion on 0.5% agar for strains wildtype DZ2 , DZ4219 (*frzS* insertion mutant) and DZ4335 (*frzS* deletion mutant). (B) Congo Red assay for EPS production shows a defect in both *frzS* backgrounds. (C) Agglutination assay in which both DZ4219 (dark green) and DZ4335 (light green) show a defect.
(TIF)
