# Supplementary figures and images for: FrzS Regulates Social Motility in Myxococcus xanthus by Controlling Exopolysaccharide Production
Source: PLoS One. 2011 Aug 19;6(8):e23920. doi: 10.1371/journal.pone.0023920 (PMC3158785; doi:10.1371/journal.pone.0023920)

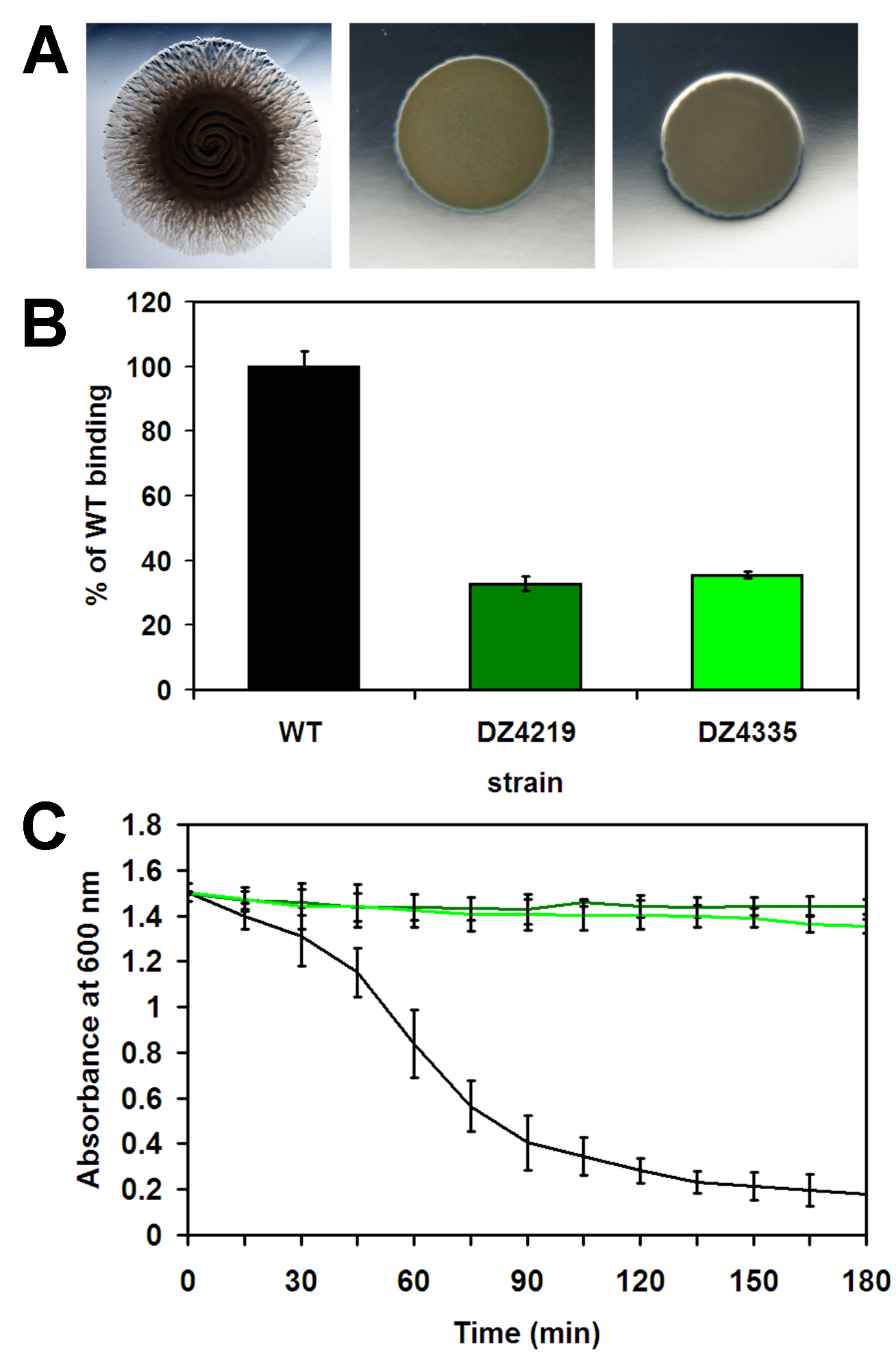

Supplement: Figure S1 — Comparison of frzS mutants. (A) From left to right, images show swarm expansion on 0.5% agar for strains wildtype DZ2 , DZ4219 (frzS insertion mutant) and DZ4335 (frzS deletion mutant). (B) Congo Red assay for EPS production shows a defect in both frzS backgrounds. (C) Agglutination assay in which both DZ4219 (dark green) and DZ4335 (light green) show a defect. (TIF) [file pone.0023920.s001.tif]
